# Activation and Molecular Targets of Peroxisome Proliferator-Activated Receptor-*γ* Ligands in Lung Cancer

**DOI:** 10.1155/2008/156875

**Published:** 2008-05-26

**Authors:** Raphael A. Nemenoff, Mary Weiser-Evans, Robert A. Winn

**Affiliations:** ^1^Division of Renal Diseases, Department of Medicine, School of Medicine, University of Colorado Denver, Denver, CO 80262, USA; ^2^Division of Hypertension and Pulmonary Sciences and Critical Care, Department of Medicine, School of Medicine, University of Colorado Denver, Denver, CO 80262, USA

## Abstract

Lung cancer is the leading cause of cancer death, and five-year survival remains poor, raising the urgency for new treatment strategies. Activation of PPAR*γ* represents a potential target for both the treatment and prevention of lung cancer. Numerous studies have examined the effect of thiazolidinediones such as rosiglitazone and pioglitazone on lung cancer cells in vitro and in xenograft models. These studies indicate that activation of PPAR*γ* inhibits cancer cell proliferation as well as invasiveness and metastasis. While activation of PPAR*γ* can occur by direct binding of pharmacological ligands to the molecule, emerging data indicate that PPAR*γ* activation can occur through engagement of other signal transduction pathways, including Wnt signaling and prostaglandin production. Data, both from preclinical models and retrospective clinical studies, indicate that activation of PPAR*γ* may represent an attractive chemopreventive strategy. This article reviews the existing biological and mechanistic experiments focusing on the role of PPAR*γ* in lung cancer, focusing specifically on nonsmall cell lung cancer.

## 1. INTRODUCTION

Lung cancer is the leading cause of cancer death for both men and women in the USA. In fact, more deaths will occur this year due to lung cancer than breast, prostate, and colorectal cancers combined [1]. In spite of intensive research, 5-year survival in patients with lung cancer remains dismally low, with overall survival at 15% [2]. A major reason for this problem is the presence of metastasis at the time of diagnosis. While smoking cessation will clearly reduce the risk of lung cancer, a majority of diagnosed cases are being detected in exsmokers [3]. Therefore, in addition to new chemotherapeutic approaches, there appears to be a critical need for chemopreventive strategies which can be administered to patients at risk for developing lung cancer. In this article, we will review recent data, both from basic sciences experiments and from clinical studies indicating that activation of the nuclear receptor peroxisome proliferator-activated receptor *γ* (PPAR*γ*) may represent a novel strategy for the treatment and prevention of lung cancer.

## 2. BIOLOGY OF LUNG CANCER

Lung cancers are categorized as small cell lung cancer (SCLC) and nonsmall cell lung cancer (NSCLC). As a group, the NSCLC constitute the bulk of lung cancers and are subdivided into squamous, adenocarcinoma, and large cell carcinoma phenotypes. Selective changes in specific oncogenes can be used to distinguish the two types of cancer. Activating mutations in ras are associated with NSCLC, with a mutation at codon 12 of the Ki-Ras gene observed in approximately 30% of adenocarcinomas, and just under 10% of other NSCLC types [4]. These mutations appear to be virtually absent from SCLC [5]. In mice, Kiras mutations are found in over 90% of spontaneous and chemically induced lung tumors [6]. Overexpression of the cmyc gene is also frequently observed in NSCLC, but appears to be more prevalent in SCLC [7]. Elevated expression of the HER-2/neu gene, a member of the epidermal growth factor receptor family has also been observed in 35% of adenocarcinomas and a slightly lower percentage of squamous carcinomas [8]. Alterations in tumor suppressor genes have also been reported. Mutations in p53 have been detected in 90% of SCLC and 50% of NSCLC [7]. Mutations in the retinoblastoma gene are more specific for SCLC, occurring in more than 90%, while only a small fraction of NSCLC have mutations in this gene. Recently, mRNA expression profiling has been used to define subclasses of lung adenocarcinoma, which can be defined by distinct patterns of gene expression [9, 10]. These studies suggest that NSCLC may in fact represent multiple diseases characterized by distinct molecular pathways. In contrast to most NSCLC, SCLC displays neuroendocrine features exemplified by the presence of cytoplasmic neurosecretory granules containing a wide variety of mitogenic neuropeptides including gastrin-releasing peptide, arginine vasopressin, neurotensin, cholecystokinin, and many others [11, 12]. Significantly, SCLC also expresses G protein-coupled receptors (GPCR) for these neuropeptides, thereby establishing autocrine-stimulated cell growth. Therapeutic strategies have targeted these neuropeptides using inhibitors of GPCRs. However, the existence of potentially redundant loops mediated by multiple neuropeptides has limited the usefulness of this strategy.

Recently, a great deal of attention has been focused on the EGF receptor, and the use of selective inhibitors of the EGF receptor tyrosine kinase (EGFR-TKI). These agents (gefitinib and erlotinib) have shown therapeutic efficacy in a subset of NSCLC patients which have somatic mutations in this receptor [13, 14]. However, responses have also been observed in patients with wild-type EGFR. Identifying strategies which would sensitize patients to EGFR-TKI therapy is under active investigation (see [15] for review).

## 3. PPAR*γ* ACTIVATION

PPAR*γ* is a member of nuclear receptor superfamily. Two major isoforms have been described, PPAR*γ*1 and PPAR*γ*2 (see [16] for review). These are splice variants, with PPAR*γ*2 being expressed predominantly in adipose tissue, whereas PPAR*γ*1 has a more widespread distribution, and is expressed in cancer cells, including lung cancer [16]. More recently a number of additional splice variants have been identified [17]. The role of these forms of PPAR*γ* remains to be established. The structure of PPAR*γ* is similar to that of most nuclear receptors; the core of the molecule consists of a DNA-binding region (DBD) and a ligand-binding region (LBD), separated by a hinge region. There are two activation domains, AF-1 at the amino terminal and AF-2 at the carboxyl terminal. The classic pathway of PPAR*γ* activation involves binding as a heterodimer with the retinoic acid X receptor to specific DNA sequences (PPAR-RE). The consensus PPAR site consists of a direct repeat of the sequence AGGTCA, separated by a single nucleotide, designated a DR-1 site. Ligand binding to the LBD causes a conformational change, which results in the release of corepressors and the binding of coactivators, resulting in increased transcription of target genes.

PPAR*γ* is activated by polyunsaturated fatty acids and eicosanoids. In particular, 15-deoxy-Δ^12,14^-PGJ_2_(dPGJ_2_) has been shown to specifically activate PPAR*γ* with micromolar affinity [18]. Lipoxygenase products of linoleic acid, 9- and 13-HODE have micromolar affinities for PPAR*γ* [19]. It is not clear whether any of these agents are actual physiologic regulators of PPAR*γ*, and a recent study has found that endogenous levels of dPGJ_2_ do not change during adipocyte differentiation [20]. Synthetic activators of PPAR*γ* include the thiazolidinediones, such as rosiglitazone and pioglitazone [21]. These compounds have insulin-sensitizing and antidiabetic activity, which is likely mediated at least in part through PPAR*γ* activation. Finally, NSAIDs, which inhibit eicosanoid production, activate PPAR*γ* albeit at higher concentrations than required for COX inhibition [22]. While all of these agents can activate PPAR*γ*, it is clear that they also stimulate “off-target” pathways which may impact their therapeutic potency [23]. Finally, it should be noted that PPAR*γ* can directly bind to other transcription factors, including NF-*κ*B and Sp1 [24]. This mechanism of action complicates the spectrum of genes that could be regulated by PPAR*γ* by engaging regulatory elements distinct from classic PPAR-RE sites [25].

## 4. CLINICAL ASSOCIATIONS WITH PPAR*γ* IN LUNG CANCER

Analysis of human lung tumors has reported that decreased expression of PPAR*γ* is correlated with a poor prognosis [26]. Further work indicated that expression of PPAR*γ* as detected by immunohistochemistry was more frequently detected in well-differentiated adenocarcinomas, compared to poorly differentiated ones. Recently, a retrospective study demonstrated a 33% reduction in lung cancer risk in diabetic patients using the TZD rosiglitazone [27]. An even more dramatic reduction was observed in African-American patients (75%). This decreased risk appeared to be specific for lung cancer, and no protective effect was observed for prostate or colon cancer. Genetic variants in the PPAR*γ* gene have been identified which are associated with a decreased risk for lung cancer [28]. These findings suggest that chemoprevention strategies using PPAR*γ* activators may be an attractive approach in patients at risk for lung cancer, and that polymorphisms in the PPAR*γ* gene may be a way to screen those patients. There are several chemoprevention trials being initiated using TZDs. However, a concern in these studies is the association of higher rates of adverse cardiac events with chronic TZD treatment, especially with rosiglitazone [29]. As discussed below, agents which target PPAR*γ* through alternative pathways may therefore represent novel therapeutic targets.

## 5. BIOLOGICAL EFFECTS OF PPAR*γ* IN LUNG CANCER CELLS

A number of studies have examined the effects of TZDs on the growth of lung cancer cells. The majority of these studies have focused on NSCLC. Administration of TZDs has been shown to inhibit growth and induce apoptosis in numerous NSCLC cell lines [30–34]. While the mechanisms for these effects are not completely understood, they appear to be mediated through both PPAR*γ*-dependent and independent effects. Induction of apoptosis may involve the tumor necrosis factor-related apoptosis-inducing ligand (TRAIL)-induced apoptosis in some cancer cell lines [35]; these effects appear to be mediated through PPAR*γ*-independent pathways. Recent studies have also demonstrated that PPAR*γ* activation induces proline oxidase, which will result in increased production of cytotoxic reactive oxygen species (ROS) [36]. Growth arrest may be mediated through induction of the cyclin kinase inhibitor p21 [37]. In this case, the mechanism of action involves PPAR*γ*-dependent induction of p21 through interactions with other transcription factors. Several studies, including work from our own laboratory have demonstrated that activation of PPAR*γ* leads to promotion of a more highly differentiated phenotype in NSCLC [32, 38]. This can be assessed by growing cells in 3-dimensional tissue culture, which has been shown to reveal epithelial features. E-cadherin is perhaps to most widely studied marker of epithelial differentiation, and both pharmacological PPAR*γ* activators and molecular overexpression of PPAR*γ* had shown increased protein and mRNA for E-cadherin. Epithelial mesenchymal transition has been associated with cancer progression and metastasis [39]. While this is still somewhat of a controversial area [40], activation of PPAR*γ* in lung cancer cells appears to inhibit invasiveness, at least in part through inhibiting or reversing EMT.

It has become evident during the past several years, that while genetic changes in cancer cells are critical for tumor initiation, progression and metastasis entail a critical contribution from the tumor microenvironment [41]. Specifically, interactions of tumor cells with vascular cells, innate immune cells, and fibroblasts control tumor angiogenesis and promote a more aggressive phenotype. These cell-cell interactions are mediated through cytokines and growth factors initially produced by the tumor cells which recruit stromal cells. Among these cytokines are factors such as MCP-1 and CCL5, critical for macrophage recruitment, and VEGF and other proangiogenic cytokines such as IL-8 which recruit vascular cells [42]. Transcriptional control of these factors is mediated by multiple transcription factors, but specifically, it has been shown that two specific factors, NF-*κ*B and HIF-1, are critical for many of these molecules. Several studies have demonstrated that PPAR*γ* activation can inhibit activation of NF-*κ*B in NSCLC [43, 44]. While effects on HIF-1 have not been documented in lung cancer cells, PPAR*γ* has been shown to inhibit HIF-1 in other systems [45]. These data indicate that activation of PPAR*γ* may disrupt communication between cancer cells and the surrounding tumor microenvironment, thus blocking progression and metastasis, distinct from antiproliferative effects on the tumor cells. In lung cancer, where metastasis has often occurred at the time of diagnosis, agents, which specifically target tumor-stromal interactions, represent a novel therapeutic approach.

## 6. UPSTREAM ACTIVATION OF PPAR*γ*

**Figure 1:**
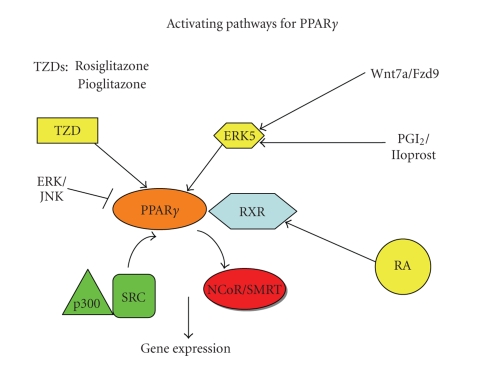
*Activation pathways for PPARγ*. PPAR*γ* forms a heterodimer with the retinoic acid X receptor (RXR). Activation can occur by thiazolidinidiones (TZD) such as rosiglitazone or pioglitazone directly binding to the ligand-binding domain. This results in the dissociation of corepressors such as NCor and SMRT, and the binding of coactivators such as p300 and Src, mediating activation of transcription. In lung cancer cells, binding of Wnt7a to its cognate receptor Fzd9 leads to activation of ERK5, which presumably directly binds to the hinge region of PPAR*γ* mediating activation. Prostacyclin (PGI) and analogs such as iloprost can also lead to PPAR*γ* activation, and this may involve ERK5 activation. Conversely, activation of the ERK or JNK family of MAP kinases can inhibit PPAR*γ* activation; this is mediated through direct phosphorylation of the molecule which alters the ligand binding affinity. Finally, activation of PPAR*γ*/RXR heterodimers may be activated through retinoic acid (RA) binding to RXR.

While TZDs have received most of the attention as PPAR*γ* activators, it is becoming apparent that activation of PPAR*γ* can occur as a consequence of activation of other signaling pathways (see Figure 1). Phosphorylation by the ERK members of the MAP kinase family has been shown to decrease PPAR*γ* activity, likely through altering the affinity for ligand binding [46]. Work in endothelial cells has demonstrated that flow-mediated activation of ERK5, a member of the MAP kinase family, results in activation of PPAR*γ* [47], which may mediate anti-inflammatory effects associated with laminar flow. In this case, the mechanism of activation involves direct binding of ERK5 to the hinge region of PPAR*γ*. In lung cancer, our studies have focused on the role of the Wnt signaling pathway. While canonical Wnt signaling has been implicated as promoting colon carcinogenesis, the role of the Wnt pathway in nonsmall cell lung cancer appears to be more complex. Our studies have demonstrated that Wnt7a signaling through its receptor Fzd9 inhibits transformed growth of NSCLC cell lines [48]. Further studies indicated that this pathway leads to increased PPAR*γ* activity through activation of ERK5, and that this increase in PPAR*γ* activity mediated the antitumorigenic effects of Wnt7a/Fzd9 signaling [49].

A connection has also been made between prostacyclin and activation of PPAR*γ*. Prostaglandin I_2_ (PGI_2_, prostacyclin), produced through the cyclooxygenase pathway via prostacyclin synthase (PGIS), is a bioactive lipid with anti-inflammatory, antiproliferative, and potent antimetastatic properties [50, 51]. Our laboratory has shown that transgenic mice with selective pulmonary PGI_2_ synthase (PGIS) overexpression exhibited significantly reduced lung tumor multiplicity and incidence in response to either chemical carcinogens or exposure to tobacco smoke [52, 53], suggesting that manipulation of the arachidonic acid pathway downstream from COX is a target for lung cancer prevention. IIoprost, a long-lasting prostacyclin analog, also inhibits lung tumorigenesis in wild-type mice. PGI_2_ can signal through a specific cell surface receptor, designated IP, which is a member of the G-protein coupled receptor family, and signals through increases in cAMP [54]. However, PGI_2_ has been shown to signal through activation of PPARs, with reports of both PPAR*γ* [55] and PPAR*δ* activation [56, 57]. To define the downstream effector of PGI_2_ in the chemoprevention of lung cancer, studies were performed in which mice overexpressing PGIS were crossed with mice deficient in IP (A. M. Meyer et al., unpublished observations). In a chemical carcinogenesis model, lack of IP did not affect protection against lung tumorigenesis mediated by PGIS overexpression, suggesting IP-independent pathways. Further study is required to whether prostacyclin can activate PPAR*γ* in vivo, and whether this effect is mediated through IP or represents a direct, IP-independent activation.

To test the role of PPAR*γ* in chemoprevention of lung cancer, we have developed transgenic mice overexpressing PPAR*γ* under the control of the surfactant protein C promoter, which targets expression to the distal lung epithelium. In a chemical carcinogenesis model, these mice showed a marked protection against developing lung tumors [44]. While the connection between prostacyclin analogs and PPAR*γ* activation needs to be more precisely defined, from a therapeutic standpoint, the ability to activate PPAR*γ* through non-TZD mechanisms represents an attractive strategy that may avoid some of the deleterious effects seen with TZD administration.

## 7. MECHANISMS OF PPAR*γ* ACTION IN LUNG CANCER CELLS

**Figure 2:**
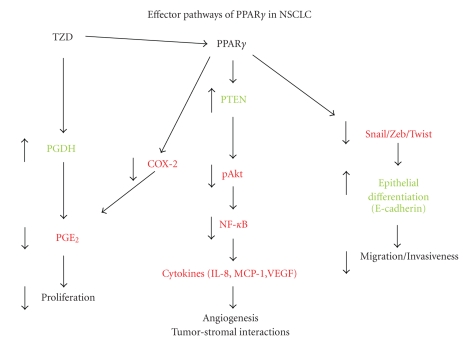
*Effector pathways for PPAR*γ* in NSCLC*. PPAR*γ* can increase either expression of enzymatic activity of PTEN. This results in inhibition of Akt activation (pAkt), which may be involved in the growth inhibitory responses seen with PPAR*γ* activation. Decreased Akt activity also can lead to decreased activity of the transcription factor NF-*κ*B. NF-*κ*B is a critical transcription factor in the production of proangiogenic and proinflammatory cytokines such as VEGF, IL-8. Decreased production of these factors would be expected to inhibit recruitment of inflammatory cells such as macrophages, and block tumor angiogenesis. PPAR*γ*-mediated suppression of members of the Snail family of transcription factors, such as Snail, Zeb, or Twist, would lead to derepression of E-cadherin expression and promote the epithelial phenotype, leading to decreased migration and invasiveness. PPAR*γ*-mediated suppression of COX-2 expression in NSCLC has been shown by several investigators. This would result in decreased PGE_2_ production, which will impact growth. TZDs can inhibit PGE_2_ production through a PPAR*γ*-independent pathway involving induction of 15-hydroxyprostaglandin dehydrogenase (PGDH). Pathways indicated in green are increased or activated by PPAR, while those in red represent pathways that are inhibited or repressed.

In spite of intensive study examining the biological effects of PPAR*γ* activation in lung cancer, much less is know regarding the direct targets of PPAR*γ* (see Figure 2). As a member of the nuclear receptor superfamily, PPAR*γ* is a ligand-activated transcription factor. Thus, one assumes that there are direct transcriptional targets, where PPAR*γ*, in combination with the RXR receptor, binds to regulatory elements and induced transcription. These targets have been difficult to identify in cancer cells. In fact, most of the responses that have been demonstrated involve suppression of target genes (e.g., cytokines). While PPAR*γ* has been shown to upregulate E-cadherin in NSCLC, there are no studies demonstrating direct binding of PPAR*γ* to the E-cadherin promoter. A family of transcription factors have been identified which act as suppressors of E-cadherin expression. Members of this family include Snail1, Snail2 (Slug), ZEB1, and Twist [58, 59] are potent inducers of EMT. Both Snail and Twist appear to play critical roles in breast cancer metastasis [60, 61]. Overexpression of ZEB-1 has been implicated in mediating EMT in NSCLC cells [62].

Several studies have reported increased expression of the protein and lipid phosphatase PTEN in response to PPAR*γ* activation [63, 64]. Increased expression/activity of PTEN would be anticipated to inhibit signaling through PI-3 kinase/Akt, and downstream effectors such as mTOR. Decreased activation of Akt could lead to inhibition of NF-*κ*B signaling [65–67], although the molecular mechanisms are not well defined.

Elevated expression of cyclooxygenase-2 (COX-2) is common in NSCLC, and mediates increased production of PGE_2_ [68]. Activation of PPAR*γ* has been shown in inhibit COX-2 expression and decrease PGE_2_ production in NSCLC [44, 69]. While the mechanisms whereby PGE_2_ contributes to growth and progression of NSCLC are not completely understood, recent data in colon cancer have shown that PGE_2_ acting through its cell surface receptor can engage *β*-catenin signaling, leading to proliferation [70]. Consistent with such a model, TZDs also inhibit expression of the EP2 receptor, which couples to *β*-catenin signaling [71]. Regulation of PGE_2_ production by TZDs can also occur through PPAR*γ*-independent pathways. Both rosiglitazone and pioglitazone can directly activate 15 hydroxyprostaglandin dehydrogenase, promoting breakdown of PGE_2_.

## 8. CONCLUSIONS AND FUTURE DIRECTIONS

Activation of PPAR*γ* appears to inhibit lung tumorigenesis at several different stages. Animal studies indicate that increased PPAR*γ* may be chemopreventive against developing lung tumors, suggesting that it can block the early stages of epithelial transformation. In established lung cancer, activation of PPAR*γ* can inhibit proliferation, induce apoptosis, and promote a less invasive phenotype through promoting epithelial differentiation, and perhaps blocking EMT. Finally, through disruption of tumor-stromal communication via inhibition of chemokine production, PPAR*γ* can negatively impact tumor progression and metastasis. These data make PPAR*γ* activators attractive agents for the treatment and prevention of lung cancer.

However, a number of significant issues remain to be resolved. In many of the studies described in this article, it is not clear if the biological responses are mediated through “on-target” activation of PPAR*γ*, or through other “off-target” effects. A strategy to address this issue is the use of molecular approaches, either overexpressing or silencing PPAR*γ* in cancer cells to complement studies with pharmacological agents. Genetic mouse models using targeted knockouts of PPAR*γ* in either cancer cells or stromal compartments will also be informative. This strategy also applies to defining the mechanisms mediating the adverse cardiovascular events reported in patients taking TZDs. Defining the molecular targets of TZDs mediating a specific response will be critical in the further development of second-generation PPAR*γ* drugs. If adverse cardiac events are mediated through “off-target” effects, then a more selective PPAR*γ* activator would be therapeutically effective, without leading to adverse cardiac events. Alternatively, if the antitumorigenic effects of TZDs are mediated through “off-target” effectors, then identifying these pathways would lead to novel therapeutic targets. Finally, the majority of studies have focused on NSCLC. Studies defining mechanisms of activation and downstream targets in SCLC are needed to determine if PPAR*γ* represents a therapeutic target for treating these forms of lung cancer.
